# Systemic aspects of conjugal resilience in couples with a child facing cancer and marrow transplantation

**DOI:** 10.3402/qhw.v11.32423

**Published:** 2016-09-28

**Authors:** Julie Martin, Katherine Péloquin, Marie-France Vachon, Michel Duval, Serge Sultan

**Affiliations:** 1 Department of Psychology, Université de Montréal, CHU Sainte-Justine, Montréal, Canada; 2 Department of Psychology, Université de Montréal, Montréal, Canada; 3 CHU Sainte-Justine, Montréal, Canada; 4 Department of Paediatrics, Université de Montréal, CHU Sainte-Justine, Montréal, Canada

**Keywords:** Paediatric cancer, parents, resilience, we-ness, systemic-constructivist couple therapy

## Abstract

**Introduction:**

The negative impact of paediatric cancer on parents is well known and is even greater when intensive treatments are used. This study aimed to describe how couples whose child has received a transplant for the treatment of leukaemia view conjugal resilience and to evaluate the role of we-ness as a precursor of conjugal adjustment.

**Methods:**

Four parental couples were interviewed. Interviews were analysed in two ways: inductive thematic analysis and rating of verbal content with the *We-ness Coding Scale*.

**Results:**

Participants report that conjugal resilience involves the identification of the couple as a team and cohesion in the couple. Being a team generates certain collaborative interactions that lead to conjugal resilience. A sense of we-ness in parents is associated with fluctuation in the frequency of themes.

**Discussion:**

Participants’ vision of conjugal resilience introduced novel themes. The sense of we-ness facilitates cohesion and the process of conjugal resilience.

Cancer is known to affect not only the patient but also every individual in his/her environment. Studies on parents confronted with child cancer highlight the levels of parental distress that are above average even long after treatment termination (Sultan, Leclair, Rondeau, Burns, & Abate, 2015). For parents, a cancer diagnosis is a real shock, and intrusive treatments, such as haematopoietic stem cell transplantation (HSCT), disrupt daily life drastically (Long & Marsland, 2011). HSCT is highly distressing for parents who often report feeling helpless, largely as a result of anticipated and actual side effects of the procedure (Oppenheim, Valteau-Couanet, Vasselon & Hartmann, 2002). Parents’ conjugal relationship may also be affected by this experience; studies also show conjugal dissatisfaction in parents, communication difficulties between partners and a weakening of the conjugal bond (Martin et al., 2014). Yet, no studies have sought to define the process through which couples adjust to this situation. A more complete comprehension of couples’ resilience in the context of childhood cancer could help professionals implement interventions to help couples cope and give their child needed support.

The systemic-constructivist approach posits that conjugal satisfaction is based on the experience of conjugal unity that implies a complex set of collaborative interactions between partners (Reid, Doell, Dalton, & Ahmad, 2008). Our literature review showed that these interactions are also responsible for conjugal resilience in the face of adversity. We define conjugal resilience as a characteristic of the couple in which conjugal satisfaction, partners’ affinity and mutual respect, and the trust they have in their relationship, are preserved or strengthened through adversity (Martin et al., 2014). In the systemic-constructivist approach, the experience of conjugal unity, which is perceived as a major resilience factor, is facilitated by the presence of a sense of we-ness. This sense of we-ness represents a personal experience leading individuals to integrate their relationship as a part of their identity, generating a sense of reciprocity between partners (Dalton, 2005; Reid et al., 2008). By experiencing this sense of we-ness, partners seek to validate their understanding of their relationship with each other and engage in verbal exchanges leading to the consolidation of a relational identity. The sense of we-ness leads partners to see themselves as essential to the couple's well-being, which incites them to work with their partner towards a better relationship functioning (Reid, Dalton, Laderoute, Doell, & Nguyen, 2006). This theory makes it possible to study how the adjustment process may unfold (e.g., what cognitive mechanisms are involved) in couples that are confronted with intense and chronic stress such as paediatric cancer. In order to test the applicability of this approach in this particular context of adversity, we designed an in-depth exploration of parental couples’ views on their resilience.

The first objective of this study was to describe how couples whose child has received a HSCT as treatment for cancer perceive their conjugal resilience. We were particularly interested in resilience processes that occurred within the couple so as to identify what aspects could be addressed in couple therapy. The second objective was to assess the role of the sense of we-ness as a precursor of conjugal adjustment.

## Method

### Participants

We recruited four heterosexual French-speaking couples (aged 36–47 years, 11–20 years of relationship) whose child successfully received a bone marrow allograft in the last 3 years for the treatment of leukaemia at a Canadian university children's hospital, from a list of 14 potential families. Inclusion criteria were living together as a couple and jointly raising the sick child. Interviews with the couples took place at their home or at the hospital, after consent forms were signed. Couples had children aged 5–19 years, three-fourths being boys. Three couples reported conjugal or family difficulties prior to diagnosis and resorted to psychosocial resources during the treatment period.

### Interview

The couple interview inquired about parents’ experience of their relationship and how they believed it was affected by their child's disease. It focused on how couples perceived their conjugal resilience. The interview was video recorded and transcribed to allow subsequent systematic analyses. Questions were adapted from the *Oral History Interview* (Buehlman, Gottman, & Katz, 1992), which focuses on the relationship history, its functioning, and its philosophy. Questions on relationship functioning in the context of cancer were adapted from Kayser, Watson, and Andrade (2007). The interview canvas can be accessed via the main author of the study.

Both partners were interviewed together because the systemic-constructivist approach requires verbal communication between partners and not only with the interviewer to be recorded (Reid et al., 2006). To make sure that both partners would feel comfortable genuinely disclosing his or her conjugal satisfaction, the interviewer actively sought the participation of both parents. The procedure was pretested for acceptability and data quality.

### Analyses

For the first objective, we used an inductive thematic analysis (ITA, Boyatzis, 1998). We categorized codes and themes associated with the experience of conjugal resilience and processes underlying resilience. ITA was conducted after each interview and themes were submitted to change after each couple was included in the analysis. For the second objective, we rated each couple's level of we-ness using the *We-ness Coding Scale* (Reid, 2000). The interrater reliability of this scale is satisfactory, and its validity is documented by close associations with self-reported measures of mutuality and intimacy (Reid et al., 2006). Parents’ results from this scale are presented in Table I. These scores were considered as variables and a link was observed between these scores and the frequency of themes emerging from the analyses (Péladeau, n.d.). This allowed us to assess the role of the sense of we-ness in the conjugal resilience process. All analyses were performed using the qualitative data analysis software *QDA Miner*. The principles of ITA and the *We-ness Coding Scale* are available in Tables II and III, respectively. Essentially, ITA helps us appreciate the content of couples’ discourse and therefore makes it possible to identify themes related to the process of conjugal resilience, whereas the *We-ness Coding Scale* helps us assess the *way* by which participants talk about their relationship, making it possible for the researcher or clinician to evaluate their respective sense of we-ness.

**Table I:** Scores on the We-ness Coding Scale.^a^

Couple	Mother	Father	Mean
01	18	17	17.5
02	16	15	15.5
03	19	19	19
04	17	17	17

*Note*: Couples 01, 03, and 04 show results superior to 16, which represent high scores and indicate that they perceive their relationship as a separate entity with its own experiences and self-interpretations. Couple 02 differs from the other three by its lower results. Its scores suggest that this couple does not present a full integration of the marital “We”, with an average score denoting the presence of an elaborate interpersonal awareness, but also of a low level of consideration of the marital unity. The parents forming this couple therefore presented a developed understanding of each partner's contribution to the welfare of their relationship, without considering their own relationship as a separate entity.

a From Reid (2000).

**Table II:** Inductive thematic analysis with QDA Miner.

Principles	
•	A method for interpreting a text content by identifying a pattern of concept
•	Imply the identification of themes categorizing discourse segments
•	Themes emerge from repeated reading and reminding of the research question
•	Categories and subcategories are determined with QDA Miner

**Table III:** The We-ness Coding Scale.^a^

Format
It consists of six principal levels of we-ness, each divided into four sub-levels (a total of 24 scores of we-ness). A higher score corresponds to a greater sense of we-ness.
How to use it
We focus on segments of relational episodes, that is, remarks made by the individuals about his/her partner or his/her couple as a unit. These segments can be descriptions of past events or exchanges between partners during the interview. A score is attributed to each relational episode by first choosing the degree and sub-degree of we-ness. The total score for each partner is the average of the scores attributed to all of his relational episodes. The degree of marital we-ness is estimated using the average of the scores of both partners (Reid et al., 2006).

a From Reid 2000.

### Quality of analyses

In order to meet quality criteria for qualitative research, we adopted several procedures (Morrow, 2005). The internal validity of the study was supported by consensus between the first author and her colleagues when performing analytical interpretations. The reproducibility of this study is permitted through the use of software that memorizes each step of the analysis. Finally, controlling for subjectivity was made possible through the use of a logbook during interviews and analyses.

## Results

### Themes on conjugal resilience

The ITA identified 36 themes pertaining to conjugal resilience (Table IV). From this list, we selected the 14 most frequent (i.e., cited by at least three couples) and representative of subcategories to be presented here, because we focus on representations shared by couples to identify the process leading to conjugal resilience. Entire results can be accessed via the main author of the study.

**Table IV:** Cataloguing of the codes following the inductive analysis.

Category	Sub-category	Code
The nature of commitment		Affinity Commitment
		Acceptance
Shared perception of the experience		Day by day Optimism
		Expectations
		Trust
		In tune
		Team other
		Mission
		Priorities
		Presence
Team	Collaboration	General collaboration
		News reporting
		Complementary
		Sharing
		Reorganization of the routine
		Care
		Confide
Dyadic coping		Limited disclosures
		Support
		Dyadic coping other
Avoidance coping		Denial
		Affirmation
		Consideration
Managing differences		Perspective taking Settlement of disputes
		Tolerance
		Distance
Maintaining the relationship		Taking care Projecting oneself
		Signs of affection
		Marital well-being
The marital resilience state		Increased trust Proximity
		Resistance

#### The definition of conjugal resilience

When asking parents to describe the impact of cancer on their relationship, they spoke of a novel sense of closeness between partners and a strengthening of the bonds connecting them. One parent said the following: “… I think we're stronger because of all that… ‘ and ’… I think we grew because of this.” It seems that emotional proximity between partners is the predominant aspect of their state of conjugal resilience.

#### The couple as a team

Parents expressed having developed a unique conjugal identity in the context of their child's cancer. Three couples reported having been more of a team than a couple in the face of this hardship. Couples set aside certain elements that would usually define their relationship so as to dedicate it exclusively to the care of the child, resulting in decreased intimacy and sexual desire, lessening of expectations that they would normally have for each other about affection, and so on. A mother elaborated on this theme in this way: “I no longer saw us as a couple. No, it's not that I no longer saw us as a couple, (…) how can I explain it (…) we were like two fighters side by side.” Although one couple did not mention this idea of a team identity, this couple reported, like the others, that they had to fulfil a common mission within the circumstances and that the parents shared the same goals and priorities in the situation. This mission principally pertained to the support they provided to the sick child through the disease. This idea came across in this way among others:

We have the same priorities too, both of us, our priority is the children and we realize that our children were raised in a crisis, that's not what we would have wanted and we're trying to compensate for that as much as we can and our focus is on that, both of us.

#### Collaborative interactions

Couples claimed that several relationship interactions contributed to the resilience of their relationship. These interactions referred to (1) the communication of medical information and effective reorganization of daily life, (2) dyadic coping for managing emotions and thoughts in relation to the hardship, and (3) strategies directly targeting the maintenance of their relationship bond and the management of discrepancies between partners.

#### Communication and reorganization of daily life

The most practical collaboration, which was particularly necessary during treatment periods for all couples, was mainly through the exchange of medical information and an efficient reorganization of the routine to accommodate the demands of the illness. When parents were asked about their mutual communication, they all reported that discussions mainly involved updates on the child's status, treatment, or medical examination results. This allowed both parents to remain informed about their child's progress and helped them establish a common understanding of the situation. One parent presented it as follows:

We talk a lot about scientific details (…), this happened today here's the update, are we understanding it the same way? Did you understand all this, it means this, it means that, we are left with this option, OK well, here's the picture.

Practical collaboration was also reflected, in all couples, by an effective reorganization of their routine. This involved organizing a relay between parents to ensure the child always had someone with him/her at the hospital and balancing tasks and responsibilities between parents. This reorganization allowed them to properly accompany their sick child while making sure to carry out other daily tasks. For example:

We have two children, one who's at the hospital, so there's always one that's here, one that's at the hospital, we try to do 50-50 (…) We would switch after lunch, we would spend an hour or two together, then after that (…), one of us would go get the other at daycare and the other would spend the evening with the other.

#### Dyadic coping

The couples described adaptive ways to manage their emotions and thoughts pertaining to their child's illness. All expressed having occasionally confided in their partner about their fears and worries concerning the child's death or the relapse of the disease. For example: “We would still talk, saying look I'm destroyed or I'm afraid (…) we still took the time to tell each other.” Nevertheless, the majority of couples (75%) specified that these disclosures were deliberately limited so as not to induce anxiety in the partner or to avoid talking excessively about the hardship. One father explained it this way:

I don't feel the need to verbalize everything all the time, because there's too much, we would just be talking about that, and as I said a little earlier, if I give in to a weakness well she needs to step up, (…) I don't want to take her there either.

All couples also talked about mutual support, mainly described as a mutual presence and non-verbal behaviours aiming to comfort each other. For example: “The little time we had together we didn't spend it bickering, we would spend it cuddling and holding hands.”

#### Strategies to maintain the couple

Parents mentioned strategies aiming to maintain their relationship or to manage differences and conflicts with each other, which contributed to their conjugal resilience. All couples stated maintaining their couple bond by making sure to dedicate time to their relationship (e.g., planning to go on dates together) and through signs of affection they would give one another. For example: “If we want it to last together, and that's what we both want (…), we have to do things together (…) we need time for the two of us together.”

Three couples also reported making efforts to limit the degree of tension regarding their way of living with the hardship. For instance, partners asserted themselves with their partner, by telling them explicitly what they liked or disliked and what their needs were in the face of this adversity. One of the mothers expressed this need of self-affirmation as follows:

I think it's the communication between the two of us, I think that's what makes the difference (…) But you know that was decided before. In the sense that yes, we would tell each other pretty much everything, but sometimes it was long before we would tell each other.

In the same vein, these couples specified that it is important when favouring marital resilience to consider the requests and needs of their partner in decision making. For example: “He would say to me: ah, that I don't like as much. OK, that's fine. I would get up and I would go. So there was always something that would accommodate the other.”

## The nature of commitment

All couples described the nature of the commitment they took when facing cancer. Two parents made an explicit commitment to one another to ensure the continuation of their relationship. The mother expressed this commitment as such: “He told me, no matter what happens, we'll stay together, you know, we'll stick together …” The other couples reported that the cohesion was established naturally between them, without having to consult each other. The effortlessness of their cohesion stemmed from their mutual affinity, an ability to understand each other without having to speak or to guess what the other was about to say. For example:

We did not need to sit down, you should do this, and I should do that, it just happened … naturally and I think that we have, you know when I say that we have a lot of affinity for each other, we just felt it.

One couple addressed this “affinity” by describing the presence of an invisible bond uniting them in times of adversity: “It's like an invisible bond (…) that will unite us forever, no matter what happens (…) It's this bond that lets us know the other is there.”

## Relationship between the we-ness score and the frequency of themes

To assess the association between the sense of we-ness and the conjugal resilience process, we examined the relationship between the frequency of themes in the couples’ speech and their average score on the We-ness Coding Scale (Table V). Below, we focus on the themes for which fluctuation in their frequency was apparently associated with scores on the We-ness Coding Scale.

**Table V:** Frequency of codes in the inductive analysis according to the average scores on the We-ness Coding Scale (increasing values).

	Mean we-ness scores in couples			
	02	04	01	03
	15.5	17	17.5	19
Proximity	3	3	6	3
Affinity	1	1	6	9
Commitment	1			
Limited disclosures	4	1	7	
Confide	3	5	4	1
Dyadic coping other	3	1	2	6
Support	2	1	2	1
News reporting	3	3	1	1
Complementary		2	5	1
Sharing		3	4	1
Preparation	8	6	5	3
Team other	4		10	5
Mission	3	2	13	9
Affirmation	7	3		2
Consideration	5	2		3
Taking care	1	2	8	6
Signs of affection	2	1		6

On the one hand, couples with a high level of we-ness tended to more frequently mention becoming a team and sharing a common mission (codes “Team other” and “Mission”). Similarly, higher we-ness scores were associated with a more frequent mention of strategies promoting the maintenance of the relationship (codes “Taking care” and “Signs of affection”). Finally, a higher sense of we-ness tended to be associated with the notion of “affinity” between partners or of an invisible bond between partners (code “Affinity”).

On the other hand, higher scores were associated with a lower need to pass on medical information (code “News reporting”). A higher sense of we-ness also seemed to be related to less frequent reporting of having to reorganize their routine (see code “Preparation”) as well as less frequent mention of how the couple managed tension between partners (see codes “Affirmation” and “Consideration”).

## Discussion

### Description of the process of conjugal resilience

The couples’ understanding of conjugal resilience was reflected by the themes identified by the ITA and how parents related one theme with the others in their speech.

After diagnosis, the majority of couples reported a spontaneous cohesion, which translated into an “invisible bond” uniting them through the hardships and allowing them to understand each other without speaking. When parents did not refer to such a bond or less frequently mentioned it, partners sought an explicit commitment to promote cohesion between them.

This cohesion, whether natural or explicit, led them to think of themselves as a team that must accomplish a mission involving common priorities and goals regarding their child's health. This shared vision of the situation and of each partner's role seemed to direct conjugal functioning towards collaborative interactions facilitating team dynamics. These interactions focused on passing on medical information between partners, an effective reorganization of their routine, dyadic coping characterized by support and limited disclosure about one's fears and worries, and various strategies for preserving conjugal bonds and managing tensions within the couple. These collaborative interactions allowed partners to achieve a state of conjugal resilience resulting principally in a strengthening of their bonds.

This description of the conjugal resilience process contains elements that go well beyond what is usually described in the current literature in paediatric oncology where parents are typically described as a fighting team. Indeed, parents evoke an “affinity,” a lived experience that stands at the basis of their natural cohesion and provides explanations to the cohesion outlined by previous studies (Patterson, Holm, & Gurney, 2004). This refers to the notion of we-ness, which involves an experience of deep understanding of the relationship.

Importantly, by stating that disclosure about illness-related fears should be limited between parents, couples seemed to refer to the co-rumination phenomenon, which corresponds to an extensive and circular discussion on a particular issue and an emphasis on the negative emotions associated with it (Rose, 2002). To our knowledge, co-rumination has not yet been studied in couple dynamics in the context of cancer. Although co-rumination yields an immediate feeling of interpersonal closeness, it is also linked to symptoms of depression and anxiety (Rose, Carlson, & Waller, 2007). This helps to understand the adaptive nature of limiting disclosure between parents in the context of their child's cancer. In fact, it has been suggested that effective parental coping in the context of cancer includes a balance between confronting and evading the disease, so as to ultimately avoid talking about it (Van Dongen-Melman, Zuuren, & Verhulst, 1998).

Finally, participants reported adaptive interactions that have scarcely been described in the paediatric oncology literature, such as the exchange of medical information between parents and strategies to preserve conjugal bonds and limit tension between them. The exchange of medical information is a logical consequence of the parents pursuing the same goals in the face of their child's illness. Indeed, passing on news to their partner concerning their child's health allows them to compensate for the physical distance between them that inevitably occurs during periods of hospitalization (Da Silva, Jacob, & Nascimento, 2010). Strategies to preserve conjugal bonds are depicted in several studies on conjugal functioning suggesting that spending quality time together is essential for conjugal satisfaction (Russell-Chapin, Chapin, & Sattler, 2001). Conflict management through assertiveness and consideration of the other's needs is also supported by the same literature (Christensen & Shenk, 1991).

### The role of we-ness in the process of resilience

Following studies from the systemic-constructivist literature, our results imply that the sense of we-ness is an important cohesion factor facilitating collaboration between partners and contributing to the implementation of conjugal adjustment processes (Reid et al., 2008). Indeed, despite the small sample size, we noted that a high sense of we-ness was associated with an ability to instinctively establish collaboration, an aspect participants attributed to their mutual affinity. This result corroborates studies showing that an increase of we-ness is associated with a better working alliance for the couple, as each partner becomes more aware of the part he or she plays within the relationship (Reid & Ahmad, 2015).

The affinity parents talked about is depicted as an invisible bond that unites them and gives them a greater sense of awareness of their relationship and of each other. This bond allows them to understand one another without having to talk and leads them to engage in reciprocal behaviours promoting teamwork. This is a verbal description of their sense of we-ness, which leads partners to validate together who they are as a couple and what is the conjugal entity they are forming. Exchanges between partners, coloured by their sense of we-ness, led to co-define their relationship (Fergus & Reid, 2001). In the context of their child's illness, this identity is that of a team, which naturally encourages them to collaborate. In the couple presenting the lowest we-ness score (Couple 02), the understanding of their couple as a team was also possible when the parents made an explicit promise to maintain their relationship through this adversity. For these parents, cohesion was thus not spontaneous but consciously established.

The observed association between themes frequency and we-ness scores also support the idea that we-ness is inherent to the conjugal resilience process. We noticed that it was more common for couples with higher we-ness levels to discuss strategies aimed towards devoting time to their relationship and expressing their affection for their partner. This can be explained by previous results suggesting that we-ness would cause the individual to become aware of the fact that their partner's well-being depends on their actions towards them. Partners are also more on the lookout for their essential role in the maintenance of the marital bonds (Reid et al., 2006).

Interestingly, higher we-ness score was also associated with a less frequent report of having to exchange medical information and reorganize the routine. Our hypothesis is that these interactions were more easily evoked in couples with a weaker sense of we-ness, as their alliance in the circumstances of the child's illness required them to worry more about their functioning than usual. In couples that spontaneously presented a strong cohesion, this type of interaction was not necessarily less frequent, but would rather occur implicitly and would not really stand out from their usual functioning (Reid et al., 2006). Similarly, couples with a lower sense of we-ness found more use in discussing strategies to manage conflicts than couples with higher we-ness levels. In fact, it has been shown that we-ness coincides with fewer disagreements between partners (Reid et al., 2008).

Finally, results suggest that we-ness is a protective factor in the event of a change in marital identity occurring in the face of adversity, by ensuring the presence of an invisible bond between parents that is equivalent to a promise to maintain their relationship. A mother explained this idea as follows:

We knew our relationship was solid, and we told ourselves (…) if we have to go through this hardship, we'll go through it together and we'll find each other afterwards.

This result implies that, even if partners have to temporarily change the way they relate to each other to adjust to the situation, a high sense of we-ness protects the essence of their relationship (Reid & Ahmad, 2015). This idea is very coherent with clinical observations indicating that a sense of we-ness facilitates the couple's adaptation to diverse life events by encouraging individuals to constantly adjust their understanding of their relationship to that of their partner's (Reid et al., 2006). In fact, we-ness is considered by researchers and clinicians as a state of symbiosis, because “it helps accepting the marriage as an ongoing forward moving process that keeps the relationship open to what life brings [partners], and secure the relationship they have.” (Reid & Ahmad, 2015)

## Limitations of the study

This study presents limitations. First, it was conducted with a small sample, which did not allow us to attain saturation. Consequently, we cannot guarantee a comprehensive presentation of the themes explaining conjugal resilience in this population (Guest, Bunce, & Johnson, 2006). Yet, our analysis is in line with our previous review, suggesting that few new themes should emerge from additional interviews (Martin et al., 2014). Second, because our sample did not include distressed couples, the themes and interpretations presented here only represent generally resilient couples and may not apply to non-resilient couples. Finally, observed associations between theme frequencies and we-ness scores could not be tested statistically because of the small sample and thus must be interpreted with caution.

## Suggestions for clinical practice

Our preliminary results suggest that health professionals consider the diagnostic value of parental couples’ state of cohesion when the child is diagnosed with cancer, as it seems to be strongly associated with the process of conjugal resilience in the face of adversity. Furthermore, because we-ness seems to facilitate conjugal cohesion, interventions promoting the development of a stronger sense of we-ness would appear indicated to foster increased relationship resilience in couples facing major stressors including a child's cancer. Therapies such as the systemic-constructivist couple therapy (SCCT) may be particularly useful to promote we-ness (Reid et al., 2008). The authors of the SCCT prescribe a set of techniques designed to improve cohesion and collaboration within the couple. These techniques could be usefully applied with parents facing high levels of adversity such as child cancer and intensive treatments such as stem cell transplant.
